# The *Anopheles coluzzii* range extends into Kenya: detection, insecticide resistance profiles and population genetic structure in relation to conspecific populations in West and Central Africa

**DOI:** 10.1186/s12936-024-04950-x

**Published:** 2024-04-26

**Authors:** Luna Kamau, Kelly L. Bennett, Eric Ochomo, Jeremy Herren, Silas Agumba, Samson Otieno, Diana Omoke, Damaris Matoke-Muhia, David Mburu, Joseph Mwangangi, Edith Ramaita, Elijah O. Juma, Charles Mbogo, Sonia Barasa, Alistair Miles

**Affiliations:** 1 Centre for Biotechnology Research and Development (CBRD), Kenya Medical Research Institute, PO Box 54840, Nairobi, 00200 Kenya; 2 Malaria Vector Genomic Surveillance, Wellcome Trust Sanger Institute, Hinxton, Cambridge, UK; 3 Centre for Global Health Research (CGHR), Kenya Medical Research Institute, Nairobi, Kenya; 4 International Center for Insect Physiology and Ecology (Icipe), Nairobi, Kenya; 5 Pwani University Biosciences Research Centre (PUBReC), Kilifi, Kenya; 6 Centre for Geographic Medicine Research-Coast (CGMR-C), Kenya Medical Research Institute, Nairobi, Kenya; 7 Ministry of Health-National Malaria Control Programme (NMCP), Kenya, Nairobi, Kenya; 8 Pan African Mosquito Control Association (PAMCA), Nairobi, Kenya; 9 Liverpool School of Tropical Medicine, Liverpool, United Kingdom

**Keywords:** *Anopheles coluzzii*, Malaria vectors, Kenya, Population structure, Insecticide resistance

## Abstract

**Background:**

*Anopheles coluzzii* is a primary vector of malaria found in West and Central Africa, but its presence has hitherto never been documented in Kenya. A thorough understanding of vector bionomics is important as it enables the implementation of targeted and effective vector control interventions. Malaria vector surveillance efforts in the country have tended to focus on historically known primary vectors. The current study sought to determine the taxonomic status of samples collected from five different malaria epidemiological zones in Kenya as well as describe the population genetic structure and insecticide resistance profiles in relation to other *An. coluzzii* populations.

**Methods:**

Mosquitoes were sampled as larvae from Busia, Kwale, Turkana, Kirinyaga and Kiambu counties, representing the range of malaria endemicities in Kenya, in 2019 and 2021 and emergent adults analysed using Whole Genome Sequencing (WGS) data processed in accordance with the *Anopheles gambiae* 1000 Genomes Project phase 3. Where available, historical samples from the same sites were included for WGS. Comparisons were made with *An. coluzzii* cohorts from West and Central Africa.

**Results:**

This study reports the detection of *An. coluzzii* for the first time in Kenya. The species was detected in Turkana County across all three time points from which samples were analyzed and its presence confirmed through taxonomic analysis. Additionally, there was a lack of strong population genetic differentiation between *An. coluzzii* from Kenya and those from the more northerly regions of West and Central Africa, suggesting they represent a connected extension to the known species range. Mutations associated with target-site resistance to DDT and pyrethroids and metabolic resistance to DDT were found at high frequencies up to 64%. The profile and frequencies of the variants observed were similar to *An. coluzzii* from West and Central Africa but the *ace-1* mutation linked to organophosphate and carbamate resistance present in *An. coluzzii* from coastal West Africa was absent in Kenya.

**Conclusions:**

These findings emphasize the need for the incorporation of genomics in comprehensive and routine vector surveillance to inform on the range of malaria vector species, and their insecticide resistance status to inform the choice of effective vector control approaches.

**Supplementary Information:**

The online version contains supplementary material available at 10.1186/s12936-024-04950-x.

## Background

Malaria is transmitted through the infectious bite of the female *Anopheles* mosquito and is a major cause of morbidity and mortality in Kenya and sub-Saharan Africa in general. In 2022, there were an estimated 249 million cases of malaria worldwide with 233 million of these occurring in the WHO African region and accounting for about 94% of all cases [1]. In Kenya, approximately 70% of the population is at risk of malaria with the disease accounting for an estimated 13–15% of outpatient consultations [2]. Despite concerted global efforts to control malaria, elimination remains a challenge in both low and high burden settings. Historically, the *Anopheles gambiae* species complex and *Anopheles funestus* group have been known to transmit malaria in Kenya. Within the *An. gambiae* complex, *An. gambiae *sensu stricto (s.s.) and *Anopheles arabiensis* were considered the major vectors, with *Anopheles merus* contributing to transmission in coastal Kenya. Invasive *Anopheles stephensi* has also recently been detected in the country, but its contribution to malaria control is yet to be evaluated [3].

*Anopheles coluzzii* is another member of the *An. gambiae* species complex, which is morphologically indistinguishable from at least ten sibling species of malaria in sub-Saharan Africa (SSA) [4, 5]. It is responsible for a significant proportion of the malaria transmission across SSA along with *An. gambiae* and *An. arabiensis*, although *An. funestus* is of increasing concern in East and Southern Africa [6]. *Anopheles coluzzii* was formally named in 2013 following accumulating evidence of subdivision between the previously described M (Mopti) and S (Savana) molecular forms of *An. gambiae,* evidence which included the presence of pre-mating barriers and genome-wide divergence and independent evolutionary trajectories; consequently, the M form was assigned the name *An. coluzzii* and the S form *An. gambiae* [7]. *Anopheles coluzzii* is widely distributed in West and Central Africa and found in sympatry with other members of the species complex and has also been documented in Somalia [8]. To date however, no record of its presence in Kenya is available. Similar to other vector species within the *An. gambiae* species complex, the distribution and role of *An. coluzzii* in malaria transmission as well as the development of insecticide resistance varies greatly in different settings [9–11].

The heterogeneities with respect to ecological characteristics and trophic habits of members of *An. gambiae *sensu lato (s.l.) have allowed the expansion of its range and contributed to its success in malaria transmission [12, 13]. Compared to *An. gambiae,* which prefers to breed in unpolluted environments typical of rural areas*, An. coluzzii* possess a greater capacity to survive in ecologically complex environments characterized by the presence of a variety of stressors. *Anopheles coluzzii* is likely to have a greater resistance to desiccation because it predominates in arid regions [14, 15]. Studies have shown *An. coluzzii* to have greater tolerance to salinity as well as xenobiotics and ammonia pollutants in larval habitat compared to *An. gambiae,* enabling extension of its range beyond traditional rural settings to densely urbanized settings [16–18]. With the trends in urban migration seen in many African countries expected to continue and unplanned human settlements in urban settings presenting a potential risk factor of increased malaria transmission [19], comprehensive vector surveillance is crucial. Such surveillance will improve the understanding of vector bionomics thereby enabling the implementation of targeted and effective vector control interventions [20–22]. These objectives are in line with the WHO Global Vector Control Response 2017–2030 strategy’s recommendation of strengthening national surveillance systems and integration with health information systems to guide vector control and effectiveness [23].

Most entomological studies and vector surveillance efforts, including those in Kenya tend to focus on the historically known primary vectors of malaria to the exclusion of unanticipated or novel vector species. Recently however, the potential role of secondary vectors in malaria transmission has been highlighted aided by the use of molecular identification tools [24, 25]. The current study used whole genome sequence (WGS) data to investigate the taxonomic relationship of samples collected across five counties in Kenya with different epidemiological parameters. This investigation describes the first report on *An. coluzzii* in Kenya, while seeking to characterize their population structure and insecticide resistance profiles.

## Methods

### Mosquito sampling, identification and rearing

**Fig. 1:**
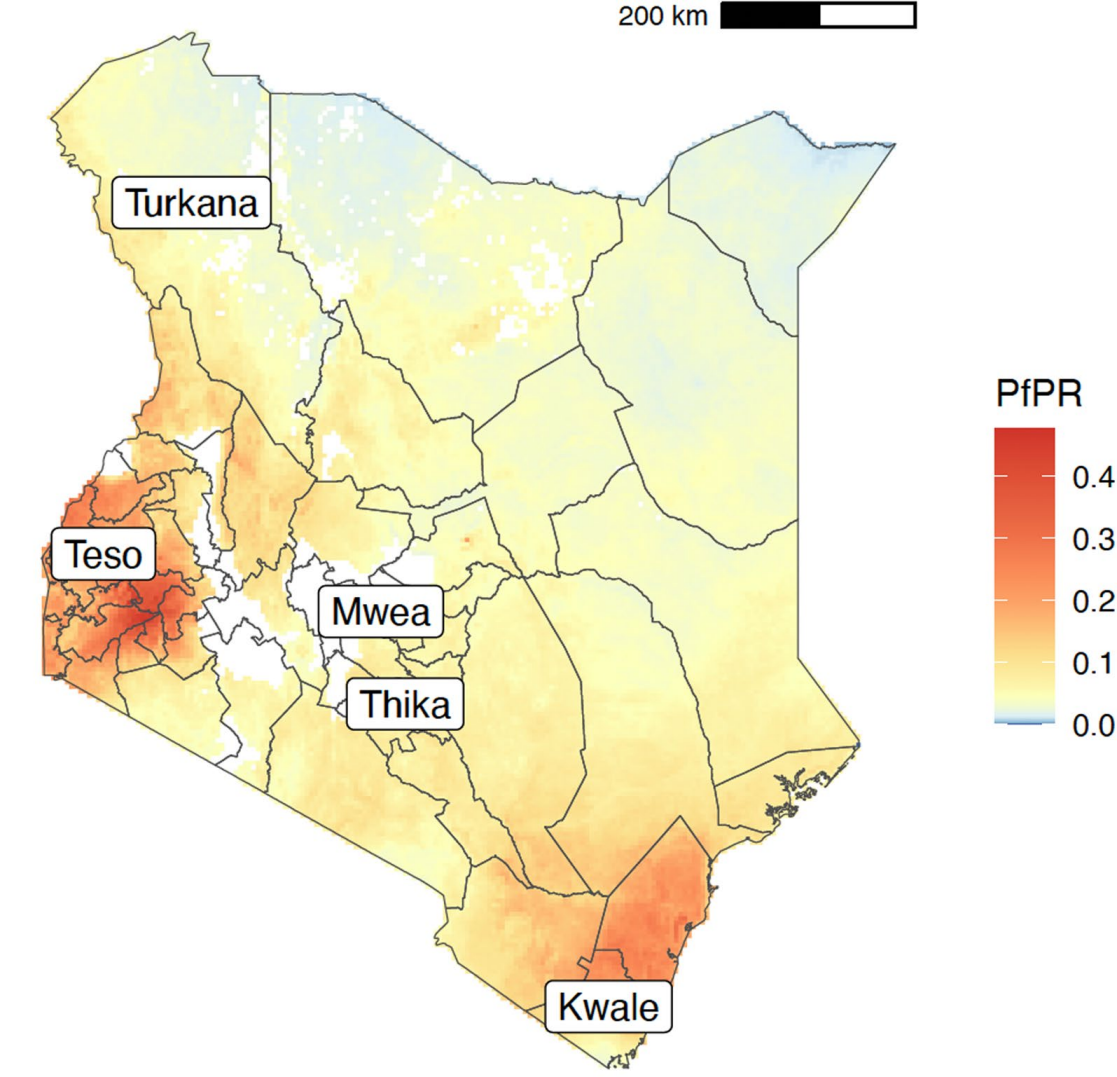
Map of Kenya showing the study sites. The figure shows the sampling locations in Kenya in relation to *Plasmodium falciparum* prevalence rate in 2015 standardized to the age group 2 to 10 years using data obtained from the malariaAtlas R package [27]

The study utilized archived mosquitoes collected from previous studies in addition to samples collected between December 2019 and February 2021 from five study sites (Fig. 1). These were: (i) Teso in Busia County, (ii) Kwale in Kwale County, (iii) Kakuma in Turkana County, (iv) Mwea in Kirinyaga County and (v) Thika in Kiambu County (Table 1). These five locations represent different ecological and malaria epidemiological zones within Kenya [26]. Teso on the western border of Kenya with Uganda is within the lake endemic zone. Kwale in the southeast is within the coastal endemic zone. Turkana in the northwest is relatively arid and within the seasonal transmission zone. Mwea and Thika are within the central highlands with Mwea being in the seasonal transmission zone and Thika in the low-risk zone.

**Table 1:** Sampling locations in Kenya and the number of samples successfully sequenced, grouped by taxon

Location	Latitude	Longitude	Year	Month	Taxon			
					*An. arabiensis*	*An. coluzzii*	*An. gambiae*	*An. quadriannulatus*
Kwale	− 4.572	39.257	2019	7	7	0	0	2
Mwea	− 0.717	37.382	2007	6	18	0	0	0
			2014	9	15	0	0	0
			2020	2	46	0	0	0
			2020	12	26	0	0	0
			2021	1	29	0	0	1
			2021	2	16	0	0	0
Teso	0.626	34.236	2013	8	24	0	4	0
			2019	7	17	0	14	0
			2019	12	43	0	17	0
Thika	− 1.061	37.181	2019	7	45	0	1	0
			2019	8	19	0	0	0
Turkana	3.717	34.857	2006	2	19	5	1	0
			2019	1	51	13	0	0
			2019	9	123	8	0	0

For both sets of mosquitoes, *An. gambiae *s.l*.* larvae identified based on morphology [28] were collected, using standard larval 350 ml dippers, from multiple breeding sites to minimize chances of sampling siblings. The larvae were transported to the laboratory for rearing to adults (except for Turkana where rearing was carried out in the field due to the long distance to the laboratory). Larvae were reared in water collected from the larval sampling site or dechlorinated tap water at temperatures between 28 °C–31 °C and humidity between 80%–85% and fed on finely ground *Sera Vipan staple diet™* (Sera, Germany) fish food. The resultant adult mosquitoes aged 2–7 days old were analysed using WGS.

### Sequencing and SNP calling

Sequencing and single nucleotide polymorphisms (SNP) calling was performed following the Ag1000G phase 3 project protocol. Briefly, paired-end multiplex libraries were prepared using Illumina’s DNA preparation protocol with fragmentation using Covaris Adaptive Focused Acoustics. Multiplexes of 12 tagged individual mosquitoes were sequenced in three replicates using Illumina HiSeq 2000 and the Illumina HiSeq X technologies. Reads were aligned to the AgamP4 reference genome using BWA version 0.7.15 and indel realignment and SNP calling performed using GATK version 3.7.0. Quality control filters applied included the exclusion of individuals with median coverage < 10X, with no coverage across > 50% of the reference genome, or samples identified as cross-contaminated by a percentage of ≥ 4.5% using the protocols set out by the AG1000G project. Only technical replicates with the best sequencing coverage were retained. Additionally, site filters defined by the Ag1000G project were applied to exclude sites where SNP calling and genotyping was less reliable because the observed genotypes were not consistent with Mendelian inheritance in laboratory crosses.

### Taxonomic assignment

To investigate taxonomic status, individual mosquitoes were assessed against two sets of ancestry-informative markers (AIMs) used to distinguish *An. gambiae* from its sister taxa *An. coluzzii* and *An. gambiae*/*An. coluzzii* from *An. arabiensis* using publicly available data from the *Anopheles* 16 genomes project [29–31]. The dataset, described by the *Anopheles gambiae* 1000 Genomes project [32], includes a set of AIMs SNPs informative in distinguishing taxa because they are exclusive to each taxonomic group discounting multiallelic sites and those with missing data. In total, 2612 and 700 AIMs were used to differentiate *An. gambiae*/*An. coluzzii* from *An. arabiensis* and *An. gambiae* from *An. coluzzii*, respectively. Individuals assessed against AIMs for distinguishing *An. gambiae* from *An. coluzzii* were called as *An. gambiae* when the fraction of coluzzii-like calls was < 0.12 and *An. coluzzii* where this fraction was > 0.9. Individuals assessed against AIMs distinguishing *An. gambiae*/*An. coluzzii* from *An. arabiensis* were called as the latter when the fraction of arabiensis-like alleles was > 0.6. Individuals in-between these fractions represent other taxa.

### Population structure

To compare the genomic composition of *An. coluzzii* in Kenya with other *An. coluzzii* cohorts, a Principal Component Analysis (PCA) dimensionality reduction on the allele counts of 100,000 biallelic SNPs equally distributed across chromosome three so as to exclude known introgressions was performed. Chosen SNPs had a minor allele frequency greater than 0.2% and no missing data. Using the same criteria for SNP selection, comparison of the evolutionary relationships of African *An. coluzzii* was carried out by constructing an unrooted Neighbour-Joining tree with a city block distance metric. To determine whether *An. coluzzii* across Africa are connected to *An. coluzzii* in Kenya, genomic differentiation between populations using Hudson’s pairwise FST was computed [33]. To further investigate whether *An. coluzzii* in Kenya have a similar demography to other *An. coluzzii*, informative summary statistics including Nucleotide diversity (θπ), Watterson's theta (*θ*_*W*_) and Tajima’s D were calculated. All analyses were performed using the open source and freely available malariagen_data python package.

### Insecticide resistance

To investigate whether *An. coluzzii* in Kenya have target site mutations associated with insecticide resistance similar to other African *An. coluzzii*, the malariagen_python package was used to calculate amino acid substitution frequencies based on the occurrence of non-synonymous SNPs at genomic sites of interest. These included the gene targeted by pyrethroid insecticides, the voltage-gated sodium channel (Vgsc; AGAP004707), the glutathione S-transferase gene conferring resistance to DDT (*Gste2*; AGAP009194), the Resistance to dieldrin gene (*Rdl*; AGAP006028) and the organophosphate target gene, acetylcholinesterase (*Ace1*; AGAP001356). To account for sequencing error and remove substitutions unlikely to be under selection, only amino acid substitutions present at a frequency greater than 5% in at least one population were retained.

## Results

### Population sampling and sequencing

A total of 1130 individual mosquitoes were collected during this study from five locations in Kenya (Fig. 1; Table 1). All locations included relatively recent sampling (2019–2021) and samples from earlier collections (2006–2014) were also available for Mwea, Teso and Turkana. A total of 744 mosquitoes with good quality extracted DNA were submitted for WGS, of which 564 passed all data quality control filters, with an average median coverage of 36 X and minimum of 10 X. After alignment to the AGAMP4 reference genome, we discovered a total of 83,052,633 SNPs segregating within the samples from this study, of which 43,701,680 passed all site quality filters previously established by the *Anopheles gambiae*1000 Genomes Project phase 3 [32].

### Taxon assignment

**Fig. 2:**
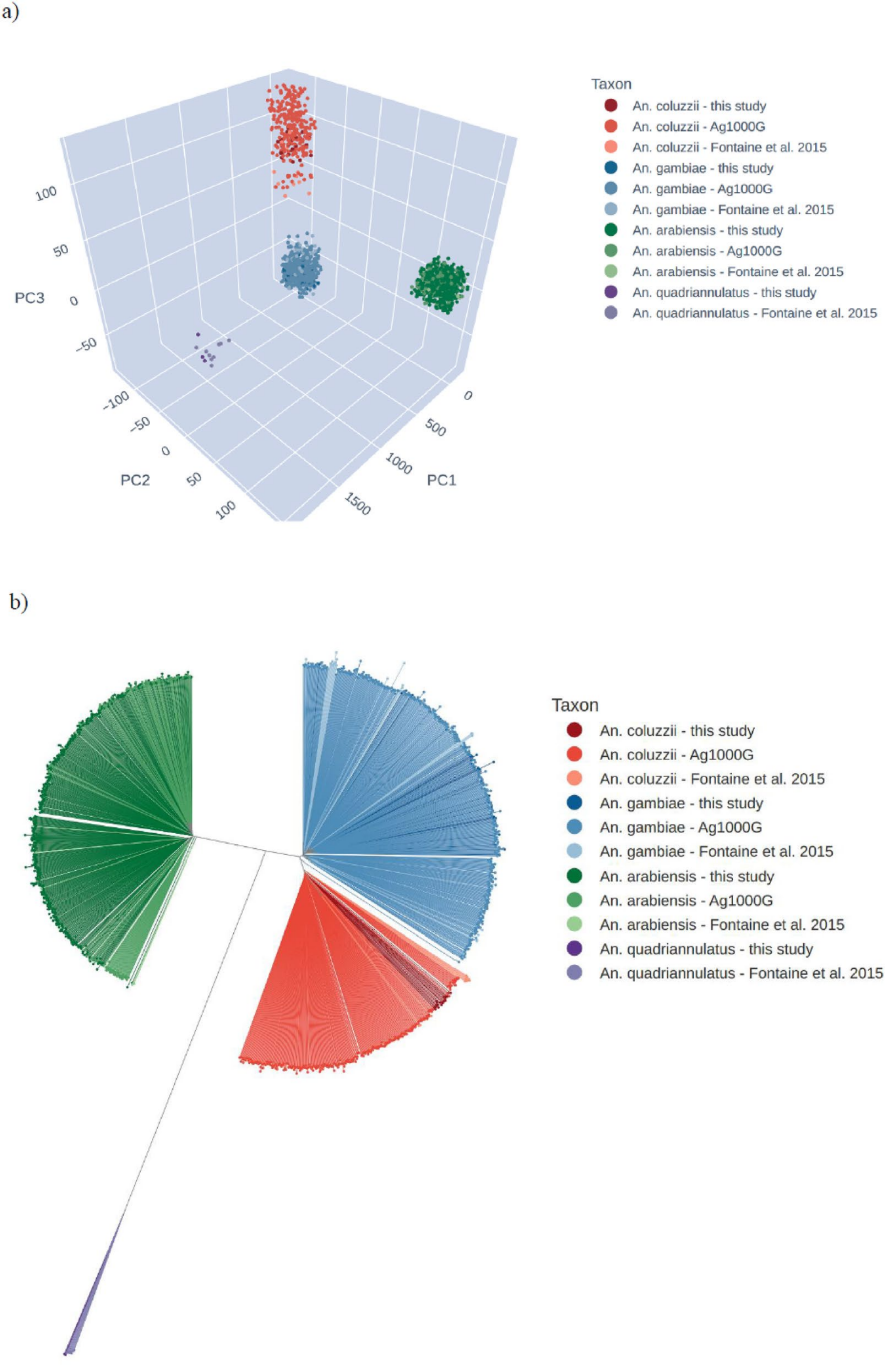
Taxon confirmation of Kenya *An. coluzzii*. Confirmation of taxon assignment using principal components analysis (PCA) and neighbour-joining tree (NJT) analysis, comparing samples in this study with reference samples from the Anopheles gambiae 1000 genomes project from inland West Africa (Burkina Faso, Mali), coastal West Africa (Cote d’Ivoire, Ghana), Central Africa (Cameroon, the Central African Republic, Angola) and the study of Fontaine et al. [31]. **a** PCA. **b** NJT

Taxon assignment within the *An. gambiae* complex is challenging because taxa are morphologically indistinguishable, and conventional genetic markers are based on a single locus that does not always reflect the ancestry of the rest of the genome [28, 29]. All samples in this study were morphologically identified as *An. gambiae* s.l*.*, then the genomic data used to investigate the species. Using a set of ancestry-informative markers (AIMs) previously ascertained from samples with known species status [30, 31], 498 samples were identified as *An. arabiensis* (arabiensis AIM fraction > 0.85; Additional file 1: Figure S1). Of the remaining samples, 37 were identified as *An. gambiae* (coluzzii AIM fraction < 0.1) and 26 were identified as *An. coluzzii* (coluzzii AIM fraction > 0.9). To provide additional confirmation of taxonomic status, a principal components analysis (PCA) was performed, and a neighbour-joining tree (NJT) constructed using genomic data from this study together with samples from other African countries from the *Anopheles gambiae* 1000 Genomes Project and the study of Fontaine et al*.* [31] (Fig. 2). These analyses showed a clear grouping by species, with the position of Kenyan *An. gambiae*, *An. arabiensis* and *An. coluzzii* samples entirely consistent with the AIM results. On comparison of data originating from multiple taxa [31], a further 3 individuals that could not be identified via AIMs were confirmed to be *Anopheles quadriannulatus* based on the PCA and NJT.

*Anopheles coluzzii* populations in West Africa are commonly found to have experienced adaptive introgression of genetic material from *An. gambiae* within a genomic region towards the centromere of chromosome arm 2L, driven by selection for pyrethroid target-site resistance alleles [35–37]. AIM profiles revealed the majority of *An. coluzzii* from Kenya are either homozygous (38%) or heterozygous (46%) for introgression from *An. gambiae* towards the centromere of 2L (Additional file 1: Figure S1).

*Anopheles coluzzii* has not previously been reported in Kenya, but is a highly competent malaria vector in West and Central Africa. In the current study, the species were detected only in Turkana and across three different sampling time points at frequencies of 20.0% (5 out of 25) in month 2 of 2006, 20.3% (13 out of 64) in month 1 of 2019 and 6.1% (8 out of 131) in month 9 of 2019. Given the importance of this finding for malaria vector surveillance and control, the remainder of this report focuses on a full characterization of the Kenyan *An. coluzzii*. Analysis of genomic data from the other *Anopheles* taxa sequenced in this study will be reported separately.

### Geographical population structure and genetic diversity

**Fig. 3:**
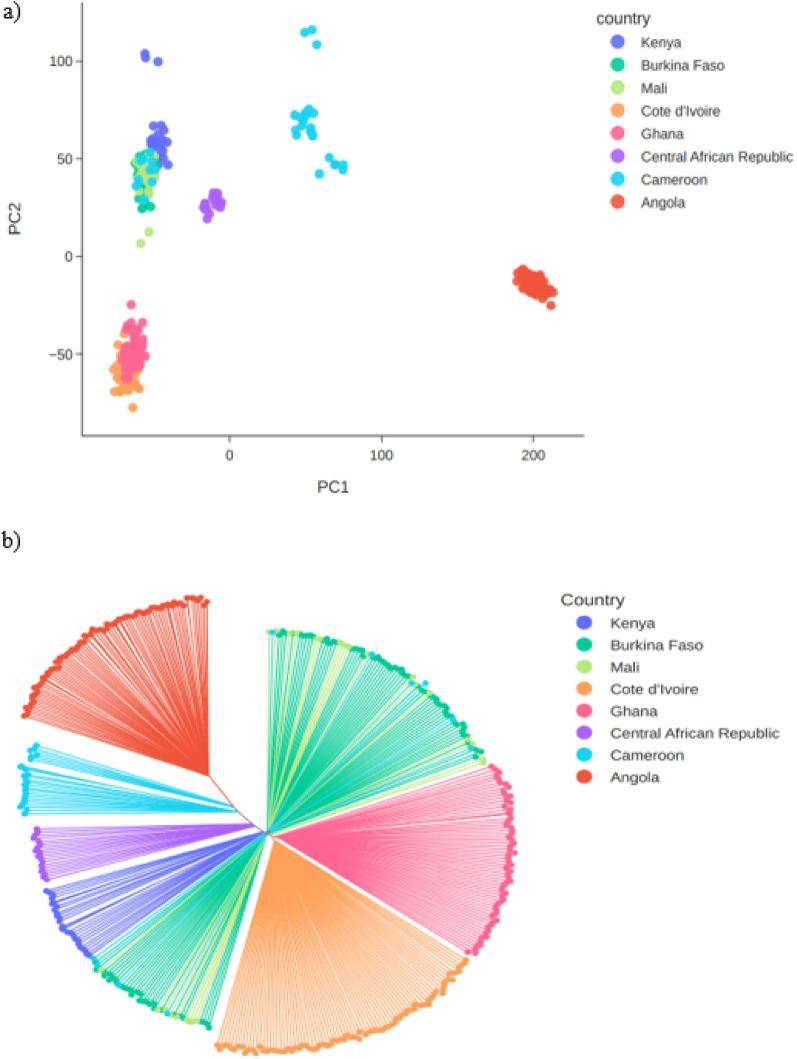
Population genetic structure of Kenyan *An. coluzzii*. The figure shows the analysis of geographical population structure within *An. coluzzii*, comparing samples from Turkana, Kenya collected in this study with reference samples from the Ag1000G project and Fontaine et al. [31]. Kenyan *An. coluzzii* are most closely related to *An. coluzzii* from inland West Africa (Mali, Burkina Faso) and Northern Cameroon. **a** PCA. **b** NJT

To explore the genetic relationship between the Kenyan *An. coluzzii* and conspecific populations from other countries, data from this study was combined with previous sequence data of *An. coluzzii* populations from inland West Africa (Burkina Faso, Mali), coastal West Africa (Côte d’Ivoire, Ghana), and Central Africa (Cameroon, the Central African Republic, Angola) [34]. Single Nucleotide Polymorphisms (SNPs) from Chromosome 3, which is free from polymorphic inversions, were used to perform a principal components analysis (PCA), compute a neighbour-joining tree (NJT) and quantify the degree of allele frequency differentiation (FST) between cohorts from different locations. The PCA and NJT analyses grouped the Kenyan *An. coluzzii* most closely with *An. coluzzii* from inland West Africa (Mali, Burkina Faso) and northern Cameroon (Fig. 3). The FST results were consistent with these analyses, finding the lowest FST between Kenya, Mali and Burkina Faso (FST 0.006–0.007 for both comparisons; Additional file 1: Table S1). These results show a lack of strong population structure between *An. coluzzii* from Kenya and more northerly regions of West and Central Africa.

To further explore whether Kenyan *An. coluzzii* share a genomic profile similar to northern West and Central Africa, a PCA targeting the 2La and 2Rb regions of the genome was performed to investigate whether the populations share the same 2La and 2Rb inversion karyotype. *Anopheles coluzzii* from Kenya have the same 2La/2Rb karyotype found in *An. coluzzii* from Burkina Faso and Mali only, supporting the finding that they are genetically most similar to these populations (Additional file 1: Figure S2). To investigate whether the Kenyan *An. coluzzii* have a similar demographic history to *An. coluzzii* populations from other countries in Africa, genetic diversity summary statistics for mosquito cohorts grouped by geographical region and year of sampling were computed. Nucleotide diversity, the density of segregating sites (Watterson’s theta) and allele frequency spectra (Tajima’s D) in Kenyan *An. coluzzii* were similar to *An. coluzzii* from West Africa (Nucleotide diversity, 0.026; Watterson’s theta, 0.033; Tajima’s D, − 0.961; Additional file 1: Figure S3), suggesting lack of genetic isolation.

### Insecticide resistance

**Fig. 4:**
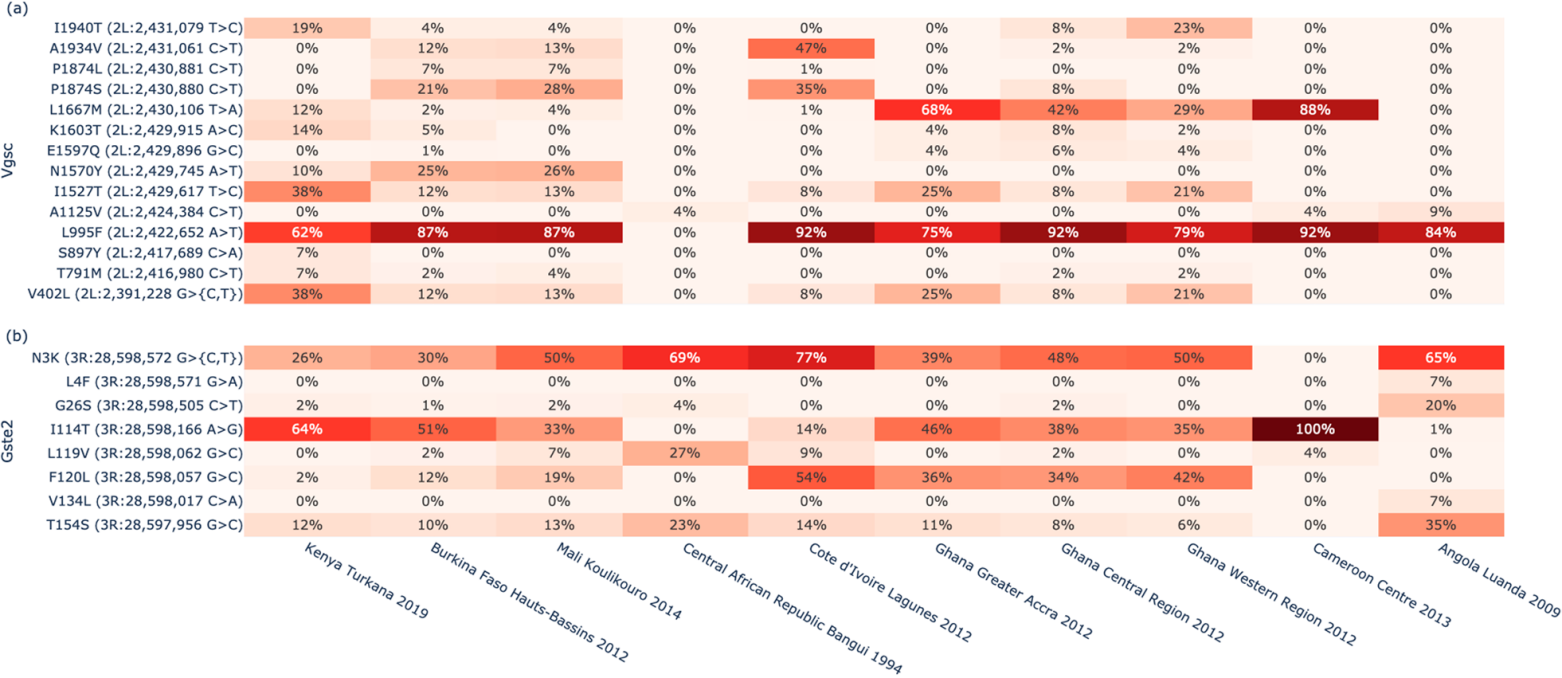
Insecticide resistance profiles of Kenyan *An. coluzzii*. The figure shows amino acid substitution frequencies in genes associated with target-site resistance to DDT and pyrethroids. **a** Vgsc (AGAP004707). **b** Gste2 (AGAP009194)

To investigate whether Kenyan *An. coluzzii* have mutations associated with target-site resistance to insecticides, we computed amino acid allele frequencies across four genes encoding insecticide binding targets for cohorts with at least ten data points: *Vgsc* (AGAP004707), *Gste2* (AGAP009194), *Rdl* (AGAP006028) and *Ace1* (AGAP001356). Kenyan *An. coluzzii* were found to have the *Vgsc-L995F* substitution associated with resistance to DDT and pyrethroids, at 62% frequency (Fig. 4a) [38]. This allele is also found at high frequency in *An. coluzzii* populations throughout West and Central Africa. In addition, Kenyan *An. coluzzii* displayed the double mutant Vgsc-V402L + I1527T substitution at 38% frequency. This double mutant is also present in other *An. coluzzii* populations. Additionally, Kenyan *An. coluzzii* have the Gste2-I114T mutation at high frequency (64%), which confers metabolic resistance to DDT [39] (Fig. 4b) and also observed at high frequency in *An. coluzzii* populations from West and Central Africa.

Two haplotypes have been previously associated with resistance to dieldrin in *An. coluzzii*, Rdl-A296G/T345M widely distributed across Africa and Rdl-A296S/T345S previously only reported from Burkina Faso [40]. The 296/345 substitution pair was observed in Burkina Faso and also in Mali and Kenya, but the frequency was very low, ~ 5%, in *An. coluzzii* from Kenya (Additional file 1: Figure S4). A mutation in the Acetylcholinesterase gene, Ace1-G280S, was previously linked to organophosphate and carbamate resistance [41]. Although the mutation is present in *An. coluzzii* from coastal West Africa (i.e., Côte d'Ivoire and Ghana), it was not present in *An. coluzzii* from Kenya (Additional file 1: Figure S5).

## Discussion

A proper understanding of vector bionomics within the context of the malaria transmission system is important for the choice and successful implementation of vector control interventions. The current study sought to improve this understanding by analyzing the distribution of *An. gambiae* s.l. sibling species from five sites in Kenya, one each in the different malaria epidemiological zones. Based on analysis of whole-genome data we report, for the first time in Kenya, the presence of *An. coluzzii*.

*Anopheles coluzzii* is a highly competent malaria vector in West and Central Africa. All the *An. coluzzii* samples found in this study were collected from Turkana in Northwest Kenya. This included mosquitoes collected across multiple years and months spanning both the dry and rainy season, confirming the presence of *An. coluzzii* in the region since at least 2006 (Table 1). Seasonal shifts in *An. gambiae* species complex composition linked to adaptation to ecology are known to occur [42, 43]. Similar to mosquito collection from Turkana, mosquitoes that were analyzed from the other sites included collections from multiple years and seasons, discounting the possibility that the failure to detect *An. coluzzii* from these sites could be related to seasonality. Past failure to detect *An. coluzzii* in Kenya is likely associated with the fact that malaria vector surveillance and identification has tended to focus on historically known primary vectors using species-specific markers. For example, in Turkana, a past study reported all mosquitoes identified as *An. arabiensis,* but only 84.8% of samples reacted in PCR diagnostic tests based on species-specific nucleotide sequences in the ribosomal DNA intergenic spacers of the *An. gambiae* species complex [44, 45]. Failure to identify specimens in such molecular assays is usually explained away as failure in the assay itself due to factors such as poor-quality DNA or other associated factors. The current analysis exploited WGS allowing the use of a large number of markers distributed across the entire genome to detect cryptic and/or unknown species demonstrating how whole genomic sequencing can be configured to support routine surveillance to accurately identify present and shifting vector distributions, adaptive evolutionary changes and populations structuring, integral to understanding of malaria transmission dynamics [46].

When the Kenya *An. coluzzii* were compared with populations from the northerly regions of West and Central Africa, they showed a lack of strong population genetic structure evidenced by clustering on the PCA and NJT and low FST, suggesting relatively unrestricted gene flow across a northerly belt spanning continental Africa. In support of this notion, Kenyan *An. coluzzii* had a similar genetic diversity to other West and Central African populations. Exploration of the 2La/2Rb inversion karyotypes, which has previously been linked to aridity and increases in frequency with this cline [47, 48] revealed that Kenyan *An. coluzzii* have the same inversion karyotype found in Burkina Faso and Mali at both loci, supporting the finding of genetic similarity to these populations. The finding that the 2La/2Rb karyotype is the same as that found in arid West and Central Africa is consistent with its environmental association, since Turkana in Kenya is arid to semi-arid, experiencing high temperatures and highly seasonal rainfall. The presence of *An. coluzzii* has also been documented in Somalia, which shares a similar arid to semi-arid ecosystem [8]. These findings suggest Kenyan *An. coluzzii* are not an isolated population, nor have they experienced any recent bottlenecks or other distinct demographic events.

Investigation of the insecticide resistance profiles of Kenyan *An. coluzzii* revealed the presence of the *Vgsc-L995F* substitution associated with resistance to DDT and pyrethroids [38]at 62% frequency and the Gste2-I114T mutation which confers metabolic resistance to DDT [39] at a frequency of 64%. These alleles are also found at high frequency in *An. coluzzii* populations from West and Central Africa [49–51]. In addition, Kenyan *An. coluzzii* displayed the double mutant Vgsc-V402L + I1527T substitution at 38% frequency also present in other *An. coluzzii* populations and recently observed to be increasing in frequency in *An. coluzzii* in Burkina Faso [52]. The V402L substitution has been functionally validated to confer insecticide resistance with reduced fitness cost to the mosquito when compared to L995F [53] and the fact that V402L is almost exclusively found in combination with I1527T suggests a strongly synergistic effect that may further increase fitness in the presence of insecticides.

Previous studies have documented resistance to pyrethroids in *An. gambiae* s.l. in Kakuma (Turkana County) with enzymatic resistance being implicated [54]. Both ITNs and IRS have historically been used to prevent malaria in the area. The finding of mutations associated with resistance to insecticides is therefore not surprising and fits with the picture of widespread and increasing insecticide resistance in the country [55]. The presence of the V402L + I1527T substitution in Kenya is concerning, because variants conferring stronger pyrethroid resistance could compromise the efficacy of new pyrethroid + PBO LLINs, currently considered among the most effective defense against resistant populations [56].

Increased urbanization promotes retention of surface water and is associated with water distribution and drainage systems that provide suitable habitat for *Anopheles* mosquitoes. The refugee camp from which the samples were collected is a highly populated region that experiences low level local transmission and has recently suffered recurrent epidemics [46]. Historically, the area has received little attention regarding malaria intervention, because the climate was considered unsuitable for known vector species, except *An. arabiensis*, considered a less competent vector.

## Conclusion

The observation that *An.* c*oluzzii*, an efficient vector of malaria which can tolerate more arid conditions and thrive in both rural and urban settings occurs in Kenya but has not been reported to date means that this vector is potentially contributing to malaria transmission in Turkana County and malaria control interventions currently in place may be ineffective against it. This finding alongside the recent finding of *An. stephensi* in Turkana and other regions of northern Kenya emphasizes the need for re-evaluation of the distribution, bionomics and epidemiological significance of the local vector populations in the country. This is the only way the country will be able to ensure vector control approaches are sufficiently targeted at the myriad of *Anopheles* vectors responsible for transmission in the different settings in Kenya.

### Supplementary Information

**Additional file 1: Table S1.** Allele frequency differentiation (FST) between different *An. coluzzii* cohorts across Africa. **Figure S1.** Taxon assignment using ancestry informative marker (AIM) genotypes. **Figure S2.** Principal components analysis of the 2La (2L:20,528,089–42,165,182) and 2Lb (2R:19,444,433–26,313,071) inversion regions to compare the karyotype of Kenyan *An. coluzzii* to other *An. coluzzii* cohorts across Africa. **Figure S3.** Bar plots of nucleotide diversity, Watterson’s theta and Tajima’s D comparing Kenyan *An. coluzzii* to other *An. coluzzii* cohorts. **Figure S4.** Amino acid frequencies for the resistance to dieldrin gene Rdl in *An. coluzzii*. **Figure S5.** Amino acid frequencies for the Ace1 gene in *An. coluzzii.*

## Data Availability

The sequences for the specimens identified in this study were submitted to The European Nucleotide Archive (ENA) (accession nos. ERR11811571–ERR11811786, ERR11812004–ERR11812101 and ERR11840239–ERR12031958).
