# Supplementary material for: The Anopheles coluzzii range extends into Kenya: detection, insecticide resistance profiles and population genetic structure in relation to conspecific populations in West and Central Africa
Source: Malar J. 2024 Apr 26;23:122. doi: 10.1186/s12936-024-04950-x (PMC11046809; doi:10.1186/s12936-024-04950-x)
Supplement: Supplementary file 1 — Additional file 1: Table S1. Allele frequency differentiation (FST) between different An. coluzzii cohorts across Africa. Figure S1. Taxon assignment using ancestry informative marker (AIM) genotypes. Figure S2. Principal components analysis of the 2La (2L:20,528,089–42,165,182) and 2Lb (2R:19,444,433–26,313,071) inversion regions to compare the karyotype of Kenyan An. coluzzii to other An. coluzzii cohorts across Africa. Figure S3. Bar plots of nucleotide diversity, Watterson’s theta and Tajima’s D comparing Kenyan An. coluzzii to other An. coluzzii cohorts. Figure S4. Amino acid frequencies for the resistance to dieldrin gene Rdl in An. coluzzii. Figure S5. Amino acid frequencies for the Ace1 gene in An. coluzzii. [file 12936_2024_4950_MOESM1_ESM.docx]

**Additional File**

**Table S1**

Allele frequency differentiation (FST) between different *An. coluzzii* cohorts across Africa.


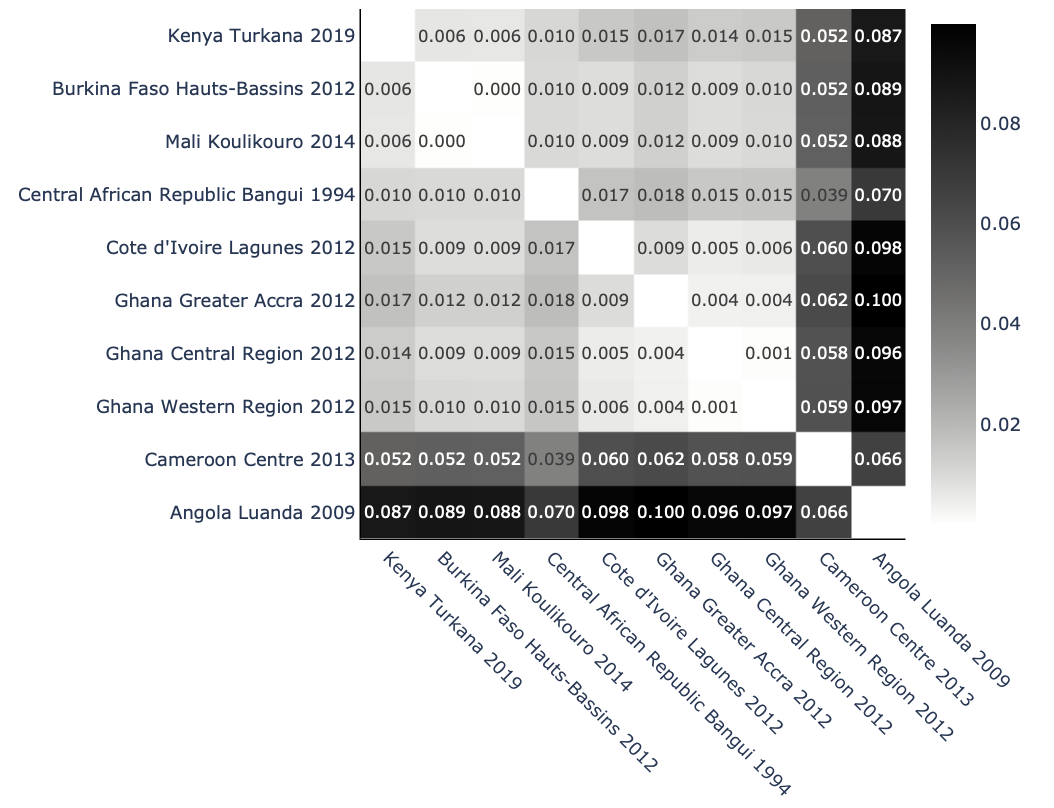


**Figure S1**

Taxon assignment using ancestry informative marker (AIM) genotypes. Plot rows represent an individual sample and columns are the AIM variants that have been genotyped. Each AIM is coloured by whether its genotype is typical of *An. arabiensis* (green), either *An. gambiae* or *An. coluzzii* (purple), *An. coluzzii* (red), *An. gambiae* (blue) or the variant is a heterozygote for the species comparison (yellow). a, Scatter plot of AIM genotype fractions, for all samples in this study. Each marker is a sample. The X axis plots the fraction of genotypes called as arab/arab in panel a. The Y axis plots the fraction of genotypes called as colu/colu in panel b, excluding chromosome arm 2L where there is a common known introgression. b, Sample genotypes at AIMs differentiating between *An. gambiae* and *An. coluzzii*, shown for all samples in this study not assigned as not *An. arabiensis.* c, Sample genotypes at AIMs differentiating between *An. arabiensis* and (*An. gambiae*, *An. coluzzii*) for samples from Turkana, Kenya.

Panel A.


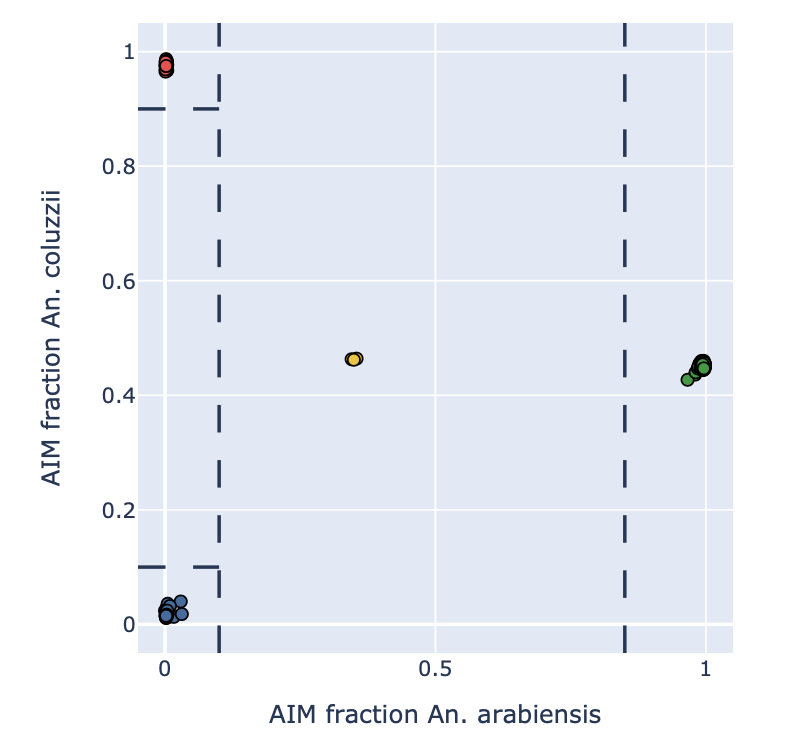


Panel B.


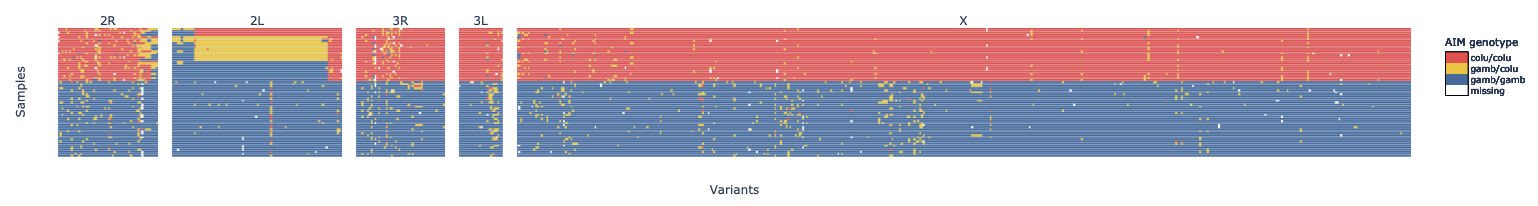


Panel C.


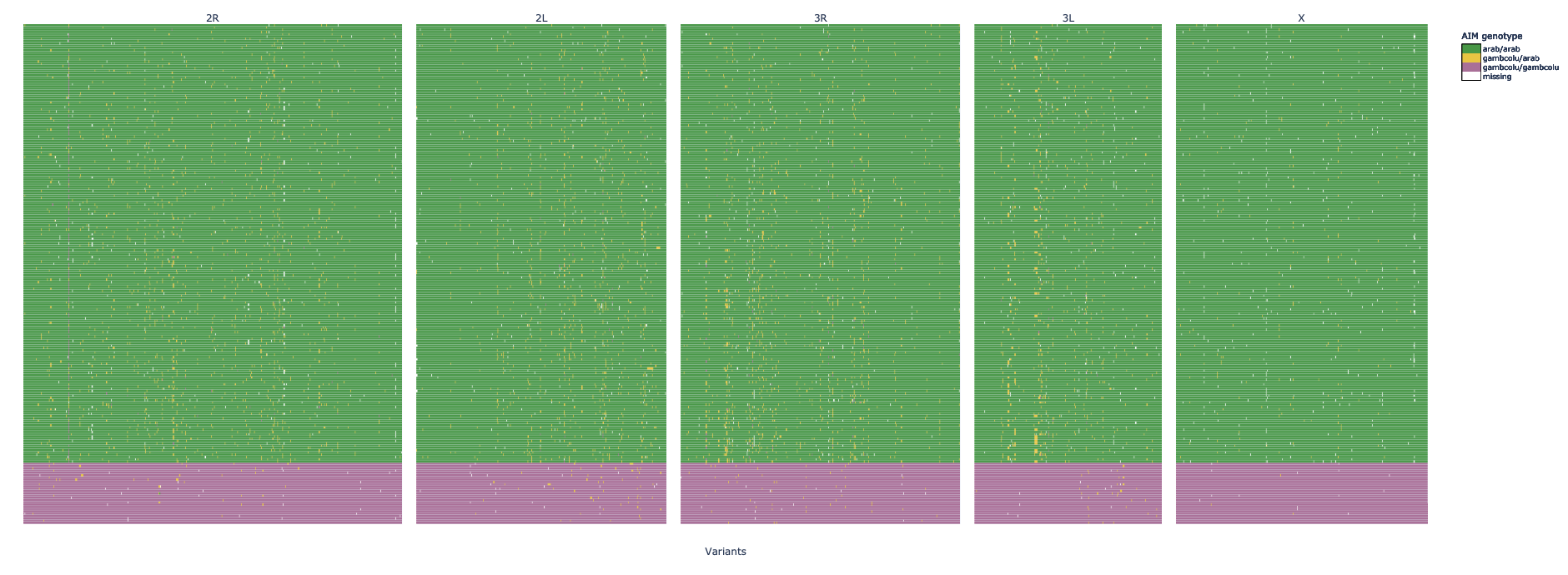


**Figure S2**

Principal components analysis of the 2La (2L:20,528,089-42,165,182) and 2Lb (2R:19,444,433-26,313,071) inversion regions to compare the karyotype of Kenyan *An. coluzzii* to other *An. coluzzii* cohorts across Africa. Principal Component 1 (PC1) from a Principal Component Analysis (PCA) within the 2La genomic region is plotted on the X axis, and the three groupings correspond to the three possible 2La karyotypes. PC1 from a PCA within the 2Rb genomic region is plotted on the Y axis, and the three groupings correspond to the three possible 2Rb karyotypes. Kenyan *An. coluzzii* cluster with *An. coluzzii* from Burkina Faso, Mali and Northern Cameroon, indicating shared karyotypes.


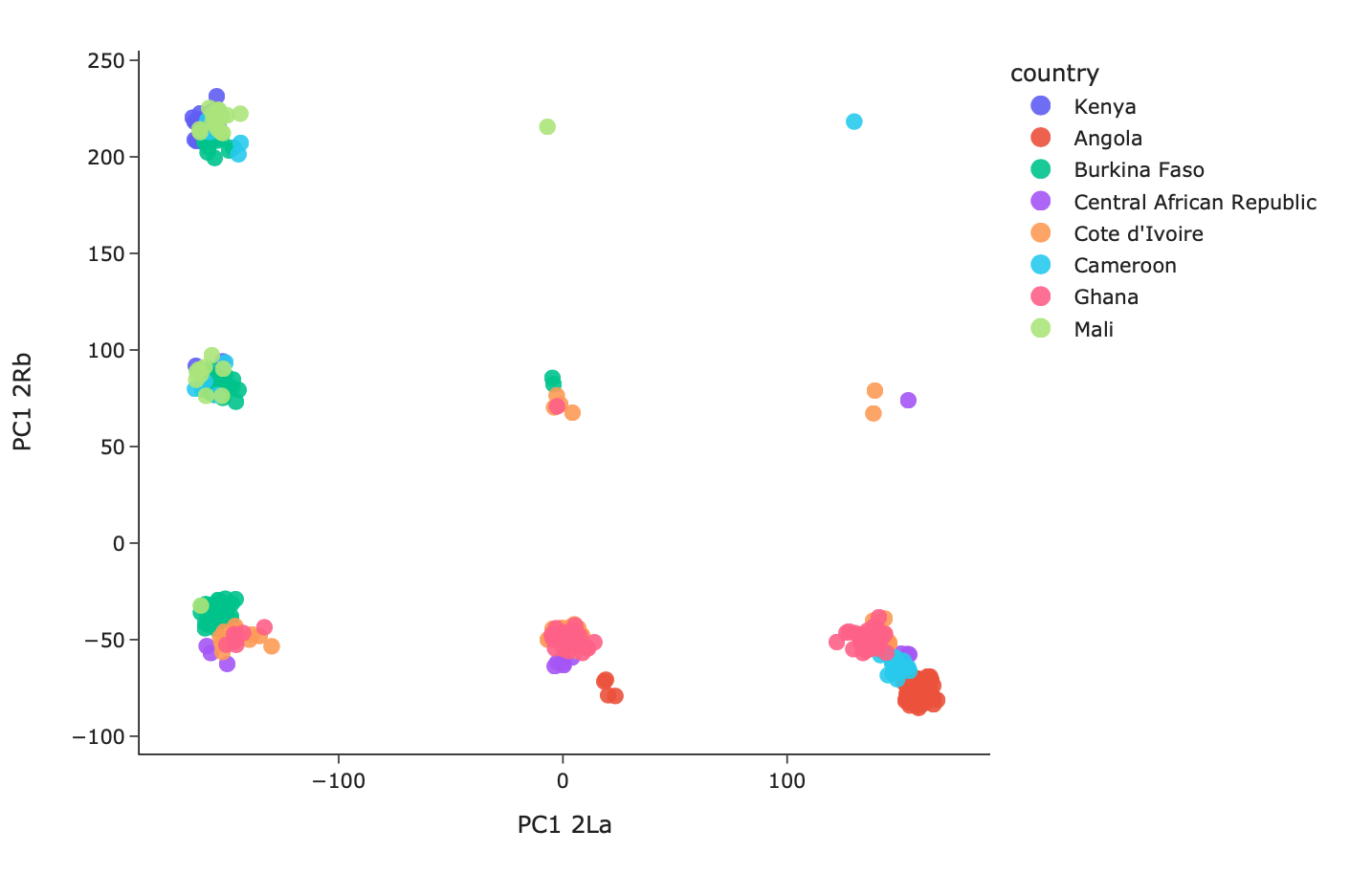


**Figure S3**

Bar plots of nucleotide diversity, Watterson's theta and Tajima's D comparing Kenyan *An. coluzzii* to other *An. coluzzii* cohorts.


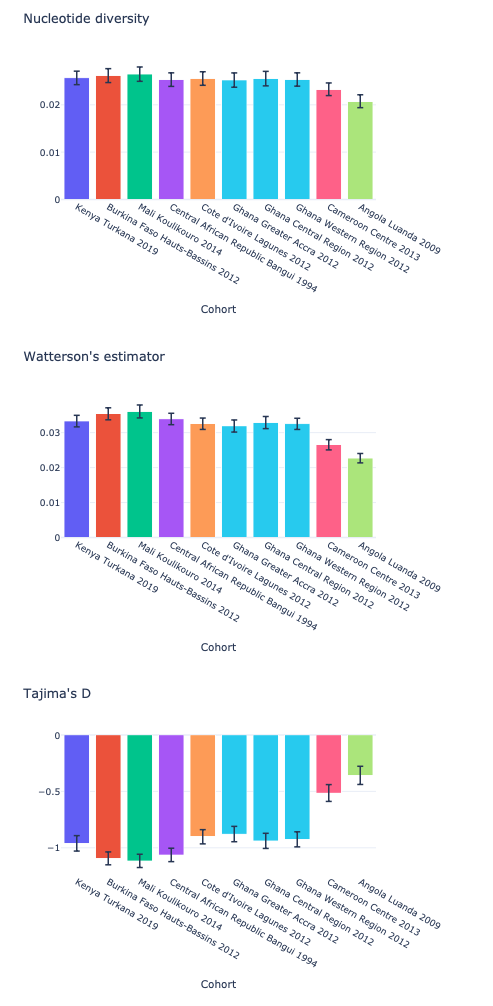


**Figure S4**

Figure S4. Amino acid frequencies for the resistance to dieldrin gene Rdl in *An. coluzzii*. The A296G/T345M and A296S/T345S substitution pairs are associated with insecticide resistance to dieldrin.


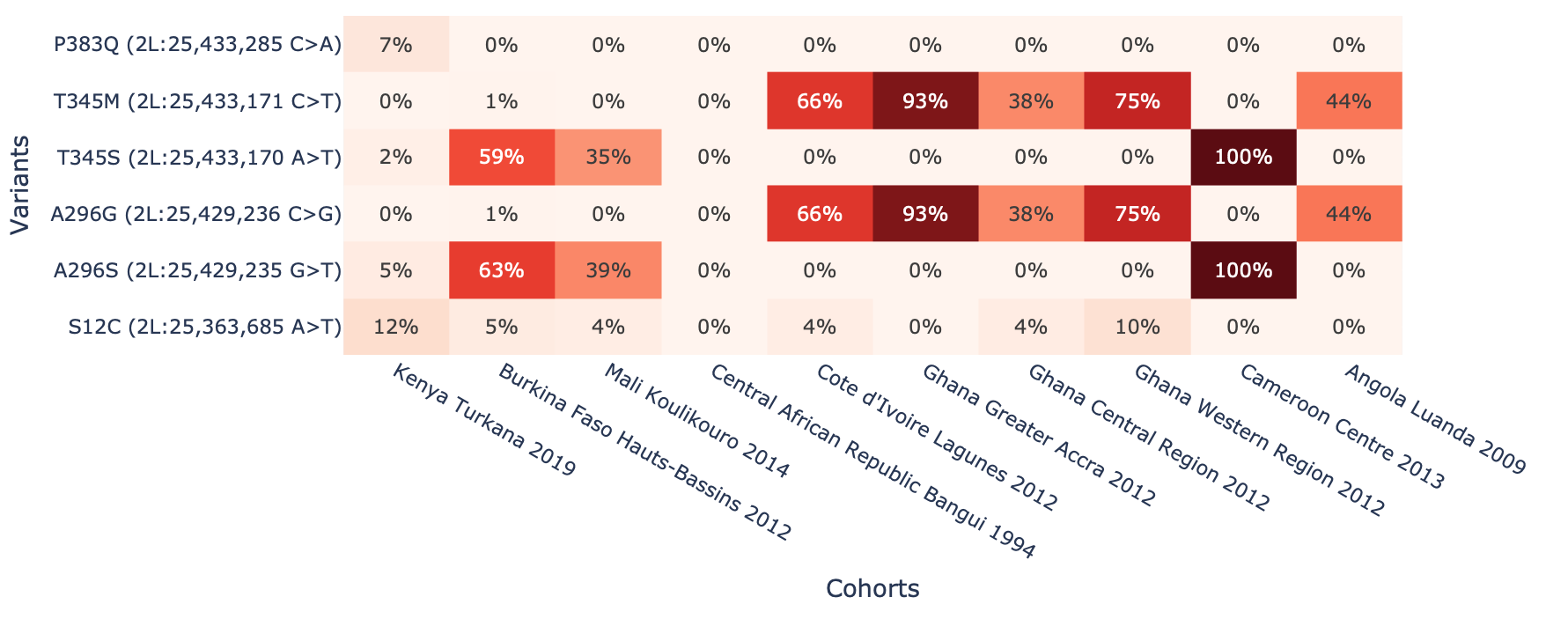


**Figure S5**

Figure S5. Amino acid frequencies for the Ace1 gene in *An. coluzzii.* The G280S substitution has been implicated in insecticide resistance to pyrethroids and organophosphates.

*
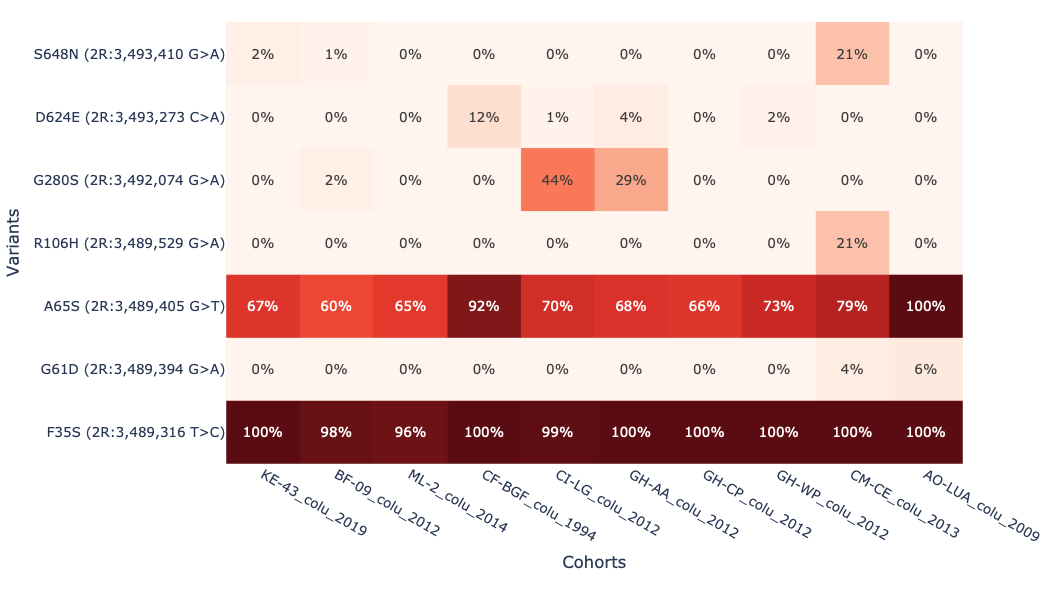
*
